# Impact of stent materials and hemodynamic changes after endovascular aneurysm repair for abdominal aortic aneurysm

**DOI:** 10.1038/s41440-026-02595-8

**Published:** 2026-03-30

**Authors:** Chih-Hsueh Tseng, Wei-Min Huang, Liang-Yin Lin, Wen-Chung Yu, Chun-Che Shih, Chern-En Chiang, Chen-Huan Chen, Shih-Hsien Sung

**Affiliations:** 1 Cardiovascular Center, Taipei Veterans General Hospital, Taipei, Taiwan; 2 Institute of Emergency and Critical Care Medicine, National Yang-Ming Chiao-Tung University, Taipei, Taiwan; 3 Division of Cardiology, Department of Internal Medicine, E-Da Hospital, I-Shou University, Kaohsiung, Taiwan; 4 Department of Internal Medicine, National Yang-Ming Chiao-Tung University, Taipei, Taiwan; 5 Department of Medicine, Kinmen Hospital, Ministry of Health and Welfare, Jinhu, Taiwan; 6 Department of Medicine, Taipei Veterans General Hospital Taoyuan Branch, Taoyuan, Taiwan; 7 Department of Surgery, School of Medicine, College of Medicine, Taipei Medical University, Taipei, Taiwan; 8 Division of Cardiovascular Surgery, Department of Surgery, Wan Fang Hospital, Taipei Medical University, Taipei, Taiwan; 9 Department of Medical Education, Taipei Veterans General Hospital, Taipei, Taiwan; 10 General Clinical Research Center, Taipei Veterans General Hospital, Taipei, Taiwan

**Keywords:** EVAR, Abdominal aortic aneurysm, cf-PWV, Morning hypertension, Implemental hypertension

## Abstract

Endovascular aortic repair (EVAR) treatment was associated with increase in arterial stiffness following the procedure abdominal aortic aneurysm (AAA). This study aims to investigate the impact of different stent-graft materials on arterial stiffness measurements and the outcomes of AAA patients undergoing EVAR. Patients with AAA undergoing EVAR were eligible for this study. Pulsatile hemodynamic parameters, including carotid-femoral pulse wave velocity (cf-PWV), carotid augmentation index (cAI), and backward pressure amplitude (Pb), were measured before and 1 month after the EVAR procedure. All-cause mortality up to 2 years post-discharge was determined by linking the study population to the National Death Registry. Among a total of 265 study participants (age 75.1 ± 11.7 years, 88.3% men), the length and diameter of the aneurysm were 7.95 ± 2.61 cm and 5.88 ± 2.08 cm, respectively. One month after EVAR, cf-PWV significantly increased, and only cf-PWV showed a significant between-group difference among stainless-steel and nitinol stent group after adjusting for MBP. At the 2-year follow-up, mortality was significantly higher in the nitinol vs. stainless-steel group and the Dacron vs. PTFE group. Furthermore, changing of cf-PWV following EVAR was an independent predictor of mortality [hazard ratio (HR) per 1-SD and 95% confidence interval (CI): 4.011 (1.154–13.950), *p* = 0.029] after accounting for confounding factors. The stent materials and fabric may influence the change of pulsatile hemodynamics after EVAR. Higher mortality rates were observed in those treated with Dacron fabric and nitinol stents, and Δcf-PWV was an independent predictor of mortality.

**Figure:**
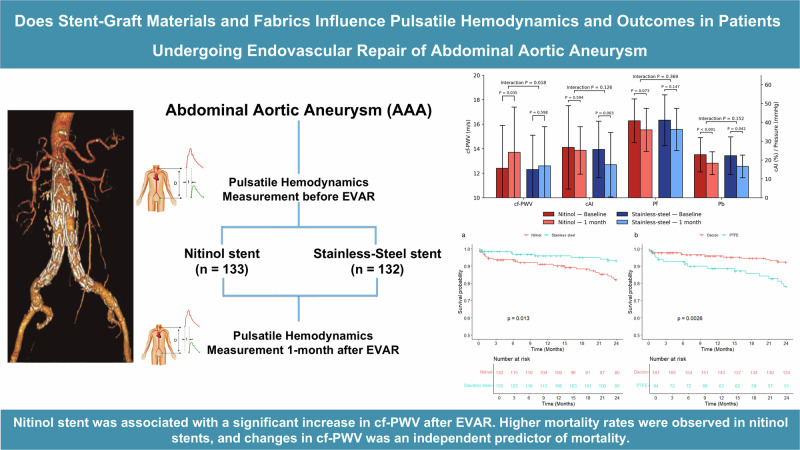
In AAA patient underwent EVAR, nitinol stent was associated with a significant increase in cf-PWV after EVAR. Higher mortality rates were observed in nitinol stents, and changes in cf-PWV was an independent predictor of mortality

In AAA patient underwent EVAR, nitinol stent was associated with a significant increase in cf-PWV after EVAR. Higher mortality rates were observed in nitinol stents, and changes in cf-PWV was an independent predictor of mortality

## Abstract (web summary)

In AAA patient underwent EVAR, nitinol stent was associated with a significant increase in cf-PWV after EVAR. Higher mortality rates were observed in nitinol stents, and changes in cf-PWV was an independent predictor of mortality.

## Introduction

Abdominal aortic aneurysm (AAA) affects up to 7.6% of the population, with a rupture mortality rate of 65.9% [1,2**].** The 2022 American Heart Association guideline of aortic disease recommends repair for symptomatic patients, those with rapidly enlarging aneurysm, or a diameter ≥5.5 cm in men or ≥5.0 cm in women [3]. Endovascular aneurysm repair (EVAR) offers lower perioperative mortality than surgical repair [4]. Factors like female sex, older age, low body mass index, and shorter aortic neck length are linked to higher long-term mortality post-EVAR [5], but the role of stent materials in post-EVAR outcomes remains unclear.

Vascular aging, reflected by arterial stiffness (carotid-femoral pulse wave velocity, cf-PWV) and wave reflections (carotid augmentation index, cAI or backward pressure wave amplitude, Pb), is a strong predictor of cardiovascular morbidity and mortality. Elevated cf-PWV has been associated with increased risks of major adverse cardiovascular events and mortality, as shown in a cohort of 442 patients with resistant hypertension [6]. Similarly, a meta-analysis of 11 studies linked higher cAI to worse cardiovascular outcomes [7]. However, in AAA patients, cf-PWV is inversely correlated with aneurysm size, potentially confounding its predictive value [8]. We previous have demonstrated cf-PWV increased and cAI decreased after EVAR, which may unmask the true vascular aging [9]. However, the role of aortic stiffness and wave reflections in predicting outcomes after EVAR remains unclear. Therefore, this study aimed to evaluate the impact of stent materials on the changes in arterial stiffness and wave reflection, and their influence on outcomes in AAA patients following EVAR.

## Methods

### Study populations

The present study enrolled patients with AAA, excluding those with acute aortic syndrome, active malignancy, or non- sinus rhythm [9]. All the study participants have undergone computed tomography. The maximal aneurysm diameter was measured perpendicular to the aortic centerline at the level of greatest dilatation inner-to-inner. The aneurysm length was defined as the longitudinal distance of the abdominal aortic segment with a diameter ≥3.5 cm along the centerline. Aneurysm neck length was also obtained. Repair was indicated according to the guidelines [3,10]. The study followed the Declaration of Helsinki principles and was approved by the Taipei Veterans General Hospital ethics committee VGHIRB No.: 2011-02-040IC and 2012-02-016AC, with informed consent obtained from all participants.

### Surgical procedures

The choice of repair procedure was determined based on the surgeon’s (C.C.S.) evaluation of the patient’s anatomical features and surgical risk. All procedures were performed electively under general anesthesia by a vascular surgeon. The endografts used consisted of either a nitinol or stainless-steel skeleton paired with stent-graft fabrics made of polyester (Dacron) or polytetrafluoroethylene (PTFE). The devices included the Medtronic Endurant (Medtronic Vascular, Santa Rosa, CA, USA; nitinol stent with Dacron fabric), Gore Excluder (W.L. Gore & Associates, Flagstaff, AZ, USA; nitinol stent with PTFE fabric), and COOK Zenith Flex (Cook Inc., Bloomington, IN, USA; stainless-steel stent with Dacron fabric).

### Echocardiography and laboratory data

Hemogram, renal function, serum electrolytes, and lipid profile were accessed by a fasting blood sample before EVAR. Estimated glomerular filtration rate (eGFR) was calculated using the Modification of Diet in Renal Disease formula, adjusted for Chinese patients [11]. Echocardiography was conducted according to the recommendations of the American Society of Echocardiography [12]. Left ventricular ejection fraction (LVEF) was measured by the biplane Simpson’s method [12]. Left ventricular internal diameter at end-diastole (LVIDd) and end-systole (LVIDs), posterior wall and interventricular septum thickness in diastole (PW and IVSd), left ventricular mass index (LVMi), and left atrial volume index (LAVi) were also obtained. Pulmonary artery systolic pressure (PASP) was estimated using continuous wave doppler.

### Measurements of pulsatile hemodynamics

Pulsatile hemodynamics were measured before EVAR and at 1-month follow-up. Patients were instructed to avoid smoking, alcohol, and caffeine for 24 h before the assessment. After resting supine for 10 min, brachial systolic blood pressure (SBPb) and diastolic blood pressure (DBPb), pulse volume recordings from arms and ankles, and pressure waveforms of the right common carotid and femoral arteries were captured simultaneously using a non-invasive vascular profiling system (VP-2000; Colin, Komaki, Japan) and tonometry probes.

Brachial pulse pressure (PPb) was calculated as the difference between SBPb and DBPb. Cf-PWV was derived from the foot-to-foot pulse transit time and the distance between the right carotid and right femoral arteries [13]. The carotid–femoral path length was measured with a non-stretchable tape from the suprasternal notch to the femoral recording site, minus the distance from the suprasternal notch to the carotid recording site, in accordance with current recommendations. Pulse transit time was determined from simultaneous ECG gating and sequential carotid and femoral waveforms. Carotid SBP was calculated as the highest point of an averaged carotid pressure waveform, calibrated using DBPb and mean arterial pressure (MAP). The inflection point, reflecting wave reflection on the carotid pressure waveform, was identified through the zero-crossing timings of the pressure waveform’s fourth derivative [13]. The computation of cAI and carotid augmented pressure (cAP) were derived from waveform analysis. The forward and backward components of carotid waveform were separated by the transit time-independent parameters using the triangulation method [14]. Forward pressure wave amplitude (Pf) and Pb represented the respective components of the carotid pressure wave. According to our previous work, the intra- and inter-observer ICCs were 0.989 and 0.988 for cf-PWV, and 0.999 and 0.984 for cAI, respectively [15].

### Follow-up

The study population was monitored through outpatient clinic visits and telephone consultations to document the adverse clinical events, including mortality and cardiovascular hospitalizations. All-cause mortality within 2 years after discharge was confirmed by cross-referencing with the National Death Registry [16].

### Statistical analysis

Continuous variables were presented as mean ± standard deviation, and categorical variables were expressed as absolute numbers and percentages. The between-group differences were analyzed using the Student’s t-test for continuous variables and the chi-squared test for categorical variables. Generalized estimating equation (GEE) were applied to analyze sequential changes in pulsatile hemodynamics and differences between stent materials and stent-graft fabrics [17]. The univariate and multivariate cox proportional hazard analyses were performed to investigate the predictors for mortality. All the analyses were conducted using IBM/SPSS v22.0 (SPSS, Chicago, IL, USA) and R-statistical software (http://www.r-project.org/). All the tests performed were 2-sided, and *p* value < 0.05 was considered statistically significant.

## Results

A total of 265patients (age 75.1 ± 11.7 years, 88.3% men) with AAA underwent EVAR. The mean aneurysm length was 7.95 ± 2.61 cm, and the mean maximal diameter was 5.88 ± 2.08 cm. Stent types were evenly distributed, with 133 patients (50.1%) receiving nitinol stents and 132 (49.9%) receiving stainless-steel stent. (Table 1) Patients treated with nitinol stent were younger, but no significant differences were observed between the two groups in sex distribution, aneurysm size, aneurysm neck length, comorbidities, cardiac structure and function, hematologic or biochemical parameters, or medication use other than renin-angiotensin system inhibitor. Regarding stent-graft fabric, 181 patients (68.3%) received Dacron stent-grafts and 84 (31.7%) received PTFE stent-grafts.

**Table 1:** Baseline characteristics of the study population, *n* = 265

	All (*n* = 265)	Nitinol stent (*n* = 133)	Stainless steel stent (*n* = 132)	*P* value
***Age (years)***	75.1 ± 11.7	72.3 ± 12.8	77.7 ± 10.1	0.005
***Male gender, n (%)***	234 (88.3)	115 (86.5)	119 (90.2)	0.551
***Vital Signs***				
SBP, mmHg	134.5 ± 18.8	135.8 ± 20.1	133.2 ± 17.39	0.375
DBP, mmHg	76.7 ± 10.5	76.7 ± 10.87	76.7 ± 10.2	0.998
Heart rate, beats/minutes	64.5 ± 10.5	65.6 ± 11.4	63.3 ± 9.4	0.090
***Co-morbidity, n (%)***				
Hypertension	218 (82.3)	107 (80.5)	111 (84.1)	0.609
Diabetes mellitus	32 (12.1)	17 (12.8)	15 (11.4)	0.689
Dyslipidemia	56 (21.1)	27 (20.3)	29 (22.0)	0.862
Smoking	115 (43.4)	65 (48.9)	50 (37.9)	0.056
Coronary artery disease	84 (31.7)	40 (30.1)	44 (33.3)	0.627
Previous myocardial infarction	14 (5.3)	7 (5.3)	7 (5.3)	0.988
Ischemic stroke	25 (9.4)	16 (12.0)	9 (6.8)	0.136
PAD	19 (7.2)	10 (7.5)	9 (6.8)	0.799
***Echocardiography***				
LA dimension (mm)	35.7 ± 7.0	35.7 ± 6.1	36.4 ± 7.4	0.862
LVIDd (mm)	50.94 ± 6.99	51.09 ± 6.78	51.30 ± 6.72	0.590
IVSd (mm)	9.77 ± 1.86	9.64 ± 1.83	9.89 ± 1.89	0.443
LVEF (%)	61.4 ± 10.7	59.1 ± 12.8	63.6 ± 9.1	0.176
LVMi (g/m^2^)	109.61 ± 32.94	111.50 ± 35.35	107.71 ± 30.51	0.517
PASP (mmHg)	31.48 ± 11.41	30.11 ± 11.34	33.22 ± 11.49	0.311
***Hemogram and Biochemistry, on Admission***				
Hemoglobin (g/dl)	12.26 ± 2.11	12.08 ± 2.08	12.43 ± 2.14	0.177
eGFR (mL/min/1.73m^2^)	61.9 ± 26.1	59.1 ± 27.4	64.6 ± 24.6	0.095
Total cholesterol (mg/dl)	168.93 ± 36.73	169.27 ± 38.22	168.60 ± 35.39	0.891
LDL cholesterol (mg/dl)	105.87 ± 33.26	105.83 ± 32.38	105.91 ± 34.22	0.986
HDL cholesterol (mg/dl)	43.12 ± 16.72	43.63 ± 16.04	42.61 ± 17.41	0.657
***Medications, n (%)***				
Beta-blocker	117 (44.2)	53 (39.8)	64 (48.5)	0.191
RAS inhibitors	88 (33.2)	36 (27.1)	52 (39.4)	0.041
CCB	142 (53.6)	64 (48.1)	78 (59.1)	0.097
Statin	30 (11.3)	16 (12.0)	14 (10.6)	0.682
***Aneurysm and treatment***				
Aneurysm diameter (cm)	5.88 ± 2.08	6.01 ± 2.18	5.71 ± 1.96	0.297
Aneurysm length (cm)	7.95 ± 2.61	7.60 ± 2.02	8.92 ± 3.68	0.141
Aneurysm neck length (mm)	30.32 ± 17.23	30.60 ± 19.21	30.06 ± 15.23	0.813
Stent diameter (mm)	30.40 ± 6.10	29.34 ± 3.86	31.02 ± 6.17	0.232
Stent length (mm)	145.40 ± 46.94	139.53 ± 29.38	151.38 ± 59.31	0.064

*CCB* calcium channel blocker, *DBP* diastolic blood pressure, *EF* ejection fraction, *eGFR* estimated glomerular filtration rate, *HDL* high-density lipoprotein, *IVSd* interventricular septum thickness at end diastole, *LA* diameter, the diameter of left atrium, *LDL* low-density lipoprotein, *LVMi* left ventricular mass index, *LVIDd* left ventricular internal diameter at end diastole, *LVIDs* left ventricular internal diameter at end systole, *RAS inhibitors* renin-angiotensin system inhibitors, *SBP* systolic blood pressure, *PAD* peripheral artery disease, *PASP* pulmonary arterial systolic pressure

**Fig. 1:**
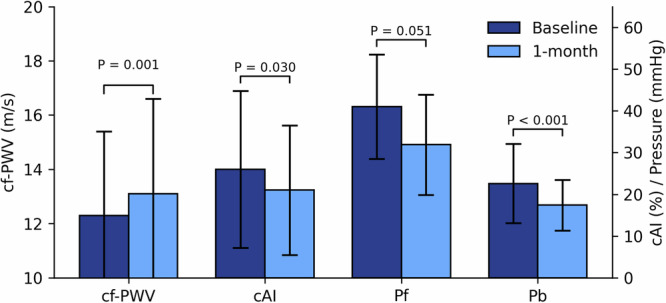
Changes in pulsatile hemodynamics at baseline and 1-month after EVAR. Parameters have been adjusted for brachial mean blood pressure by generalized estimation equation. cAI carotid augmentation index, cf-PWV carotid–femoral pulse wave velocity, EVAR endovascular aneurysm repair, Pb backward pressure wave amplitude, Pf forward pressure wave amplitude

**Fig. 2:**
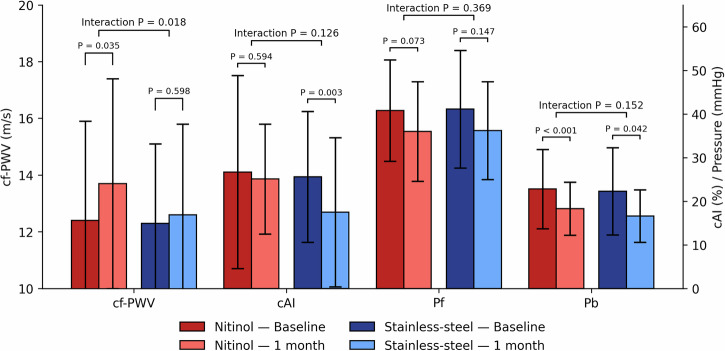
Changes in pulsatile hemodynamics across different stent materials at baseline and 1-month after EVAR. Parameters have been adjusted for brachial mean blood pressure by generalized estimation equation. Interaction p represents the stent material between-group differences. cAI carotid augmentation index, cf-PWV carotid–femoral pulse wave velocity, EVAR endovascular aneurysm repair, Pb backward pressure wave amplitude

**Fig. 3:**
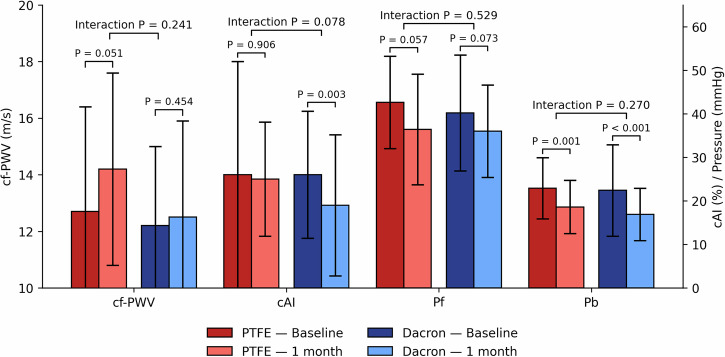
Changes in pulsatile hemodynamics across different stent-graft fabrics at baseline and 1-month after EVAR. Parameters have been adjusted for brachial mean blood pressure by generalized estimation equation. Interaction p represents the stent-graft fabrics between-group differences. cAI carotid augmentation index, cf-PWV carotid–femoral pulse wave velocity, EVAR endovascular aneurysm repair, Pb backward pressure wave amplitude, Pf forward pressure wave amplitude, PTFE polytetrafluoroethylene

At one month after EVAR, cf-PWV and Pf significantly increased (cf-PWV 12.3 ± 3.1 to 13.1 ± 3.5 m/s; Pf 40.5 ± 11.0 to 42.8 ± 12.6). In contrast, Pb decreased significantly (21.9 ± 7.3 to 20.5 ± 7.3) (Table 2). In the GEE model, post-EVAR on-treatment cf-PWV significantly increased, and cAI, Pb decreased after adjusting for MBP, while Pf showed no significant change (Fig. 1). In analyses of different stent materials adjusted for MAP, stainless-steel stents were associated with a smaller increase in cf-PWV compared with nitinol stents (interaction *p* = 0.018), whereas changes in cAI, and Pb did not differ significantly between stent materials (Fig. 2). For stent-graft fabrics, Dacron stent-grafts were associated with significant reductions in cAI and compared with PTFE, but the between group difference of the stent-graft fabric was insignificant. Cf-PWV changes were similar between groups (interaction *p* = 0.241). Pb decreased in both Dacron and PTFE groups (Fig. 3).

**Table 2:** Differences of pulsatile hemodynamics before and after EVAR

	AAA subjects receiving EVAR		
	Baseline	1-month follow-up	*P* value^*^
cf-PWV (m/s)	12.34 ± 3.11	13.07 ± 3.49	0.005
cAI (%)	26.0 ± 18.8	21.0 ± 15.5	0.142
Pf (mmHg)	40.5 ± 11.0	42.8 ± 12.6	< 0.001
Pb (mmHg)	21.9 ± 7.3	20.5 ± 7.3	0.002

*Analysis using paired t-test

*cAI* carotid augmentation index, *cf-PWV* carotid–femoral pulse wave velocity, *EVAR* endovascular aneurysm repair, *Pb* backward pressure wave amplitude, *Pf* forward pressure wave amplitude

**Fig. 4:**
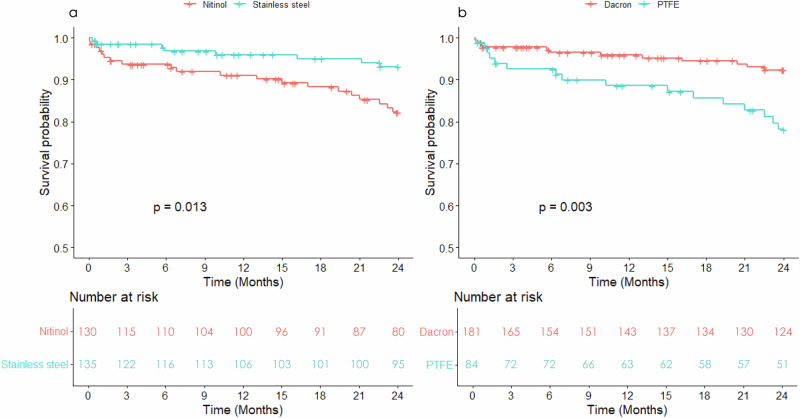
Kaplan-Meier analysis of 2-year all-cause mortality in EVAR subjects. The Kaplan–Meier survival curve analysis of **a** 2-year all-cause mortality regarding stent materials **b** 2-year all-cause mortality regarding stent-graft fabrics. PTFE polytetrafluoroethylene

During a 2-year follow-up duration, 25 patients (9.4%) died. Kaplan–Meier survival curves showed higher mortality in patients treated with nitinol compared with stainless-steel stents (log-rank *p* = 0.013) and in those with PTFE compared with Dacron stent-grafts (log-rank *p* = 0.003) (Fig. 4). In addition, nitinol stents had a higher incidence of endoleak, mainly driven by type II endoleaks, whereas the reintervention rate was similar between the two stent groups (Supplementary Table 1).

The univariate Cox hazard model showed that the aneurysm diameters on-treatment cf-PWV, change in cf-PWV (Δcf-PWV) were positively correlated with mortality, while eGFR, nitinol stent material of, PTFE stent-graft fabric and baseline cf-PWV were negatively correlated with mortality. (Supplementary Table 2)

In multivariate Cox regression analysis, the Δcf-PWV following EVAR independently predicted 2-year mortality [hazard ratio (HR) per 1-SD and 95% confidence interval (CI): 5.118 (1.784-14.687), *p* = 0.002] after adjusting for age, and gender. (Supplementary Table 3**, Model 1**). With further adjustments for age, gender, aneurysm diameter and baseline cf-PWV, Δcf-PWV and on-treatment cf-PWV were both significant predictors for mortality. [HR and 95% CI: 4.011 (1.154–13.950), p = 0.029 and 3.373 (1.133–10.042), *p* = 0.029, respectively] (Supplementary Table 3**, Model 2**) Adjustment for age, gender, body mass index, hypertension, diabetes, dyslipidemia, stent length, and smoking, Δcf-PWV remained independent predictor for mortality. (Supplementary Table 3**, Model 3**) The full multivariable regression model of change in cf-PWV was showed in table S4. Mediation analysis showed Δcf-PWV remained a predictor of mortality with significant direct effect and insignificant indirect effect mediated by stent material (Supplementary Fig. 2).

## Discussion

This study evaluated the impact of stent materials and stent-graft fabrics on pulsatile hemodynamics and outcomes in patients with AAA undergoing EVAR. We demonstrated that cf-PWV increased, while cAI, cAP, and Pb decreased following EVAR in patients with AAA. Among these parameters, cf-PWV showed a significant difference between stent materials, with stainless-steel stents mitigating the increase in cf-PWV compared to nitinol stents after adjusting for MAP. Dacron fabric also exhibited favorable hemodynamic effects, avoiding an increase in cf-PWV while significantly reducing cAI, cAP and Pb compared to PTFE fabric. The results suggested the potential role of stent material in modulating vascular stiffness beyond aneurysm exclusion. Furthermore, the stainless-steel stents and Dacron fabric was associated with better long-term survival compared to nitinol stents and PTFE fabric. After adjusting for age, gender, aneurysm diameter and baseline cf-PWV, Δcf-PWV after EVAR emerged as an independent predictor of mortality.

### Wave reflection and arterial stiffness in AAA subjects

The measurement of cf-PWV as an index of arterial stiffness is based on the Moens-Korteweg equation, which assumes uniform vessel cross-sectional area and wall thickness [18]. Because cf-PWV is inversely related to vessel diameter, the presence of an AAA generally leads to a lower cf-PWV compared with matched cohorts. Kadoglou et al. showed in a cohort of 48 AAA patients that cf-PWV increased significantly after EVAR procedure [19]. The present study also showed that cf-PWV increases after EVAR even after adjustment for MBP. Wave reflection indices, such as cAI and Pb, which are closely linked to pressure wave velocity, also provide important insights. Swillens et al., using a simulation model, showed that AAA increases reflection wave amplitude, which normalizes after EVAR [20]. Georgakarakos et al. reported nonsignificant decrease in cAI after EVAR [21], while Valdivia et al. observed a significant reduction in cAI in 44 patients after either EVAR or open surgical repair of AAA [22]. Consistent with these findings, our study demonstrated significant decreases in cAI, and Pb after EVAR, independent of MBP adjustment. EVAR excludes the aneurysmal segment and alters aortic geometry and wall properties through implantation of a stent-graft system, resulting in an increase in effective proximal aortic stiffness. This change is expected to augment characteristic impedance and Pf. Simultaneously, exclusion of the aneurysm reduces impedance mismatch and wave reflection from the abdominal aorta, leading to lower Pb and cAI. These changes are therefore physiologically concordant and reflect altered aortic impedance and improved wave transmission rather than impaired ventricular function.

### Stent material and post-EVAR arterial stiffness

Stainless steel and nitinol are the most common stent materials used in EVAR. Both provide good formability, biocompatibility, and structural strength. Compared to stainless steel, nitinol exhibits two- to fourfold higher tensile strength and excellent corrosion resistance [23]. Valdivia et al. demonstrated in a cohort of 25 AAA patients treated exclusively with nitinol stents, reported a significant reduction in cAI at 4-6 weeks after EVAR, while carotid-radial PWV remained unchanged [22].

In the present study, cf-PWV increased significantly in the nitinol group but not in the stainless-steel group after EVAR. A decrease in cAI was observed in the stainless-steel stent group but not in the nitinol stent group, whereas Pb decreased in both groups. Notably, only cf-PWV showed a significant interaction between the stent material after adjustment for MBP. These findings suggest that nitinol stents are associated with a greater increase in arterial stiffness compared with stainless-steel stents. Given nitinol’s higher tensile strength, greater chronic outward force on the vessel wall may contribute to this effect. Importantly, this was not driven by peripheral arterial stiffness, as indicated by the similar patterns of change in cAI and Pb between groups.

### Stent-graft fabrics and post-EVAR arterial stiffness

Human vessels possess viscoelastic properties that buffer pulsatile sheer stress, reducing injury to the vessel wall and limiting inflammation and remodeling. In contrast, the prosthetic stent-grafts commonly used in EVAR, PTFE and Dacron, are considerably more rigid and may contribute to increased vascular stiffness [23]. Although their tensile strengths are similar, PTFE offers greater biocompatibility and density but less abrasion resistance compared with Dacron [23]. Sartipy et al. reported a cohort of 69 AAA subjects underwent EVAR that Dacron fabric stent-graft was associated with higherpost-operative c-reactive protein and procalcitonin, as well as longer hospitalizations, compared with PTFE [24]. Similarly, Kadoglou et al. studied 118 AAA patients and found that Dacron was associated with a greater increase of interleukin-8 and osteoprotegerin, though c-reactive protein was comparable to PTFE [25]. These findings suggest that Dacron may trigger a stronger pro-inflammatory response, consistent with its lower biocompatibility. Kadoglou et al. also observed a significant increase in PWV after EVAR [25], but their analyses did not adjust for MBP, and no direct link between inflammation and arterial stiffness or wave reflection was established. In our study of 265 EVAR subjects, a larger cohort than previous reports, cf-PWV and other pulsatile hemodynamic changes were comparable across PTFE and Dacron groups after adjusting for MBP, suggesting that any fabric-related pro-inflammatory effects may not directly translate into differences in arterial stiffness.

### Stent materials, stent-graft fabrics and mortality after EVAR

Several studies have explored predictors of mortality after EVAR. Ozen et al. found that the Glasgow Aneurysm Score, including age, myocardial, cerebrovascular, and renal disease, predicted 2-year mortality [26]. Torabi et al. in 154 EVAR patients, found that only age and anemia independently predicted 5-year mortality [27]. In the present study, we found that patients treated with nitinol stents or PTFE stent-grafts had higher 2-year mortality compared with those receiving stainless-steel stents or Dacron stent-grafts. Moreover, baseline and on-treatment cf-PWV and ∆cf-PWV but not wave reflection indices were associated with mortality. After accounting for confounders, ∆cf-PWV remained independent related to mortality. The prognostic value of cf-PWV has been widely established in the general population, particularly for cardiovascular events and all-cause mortality [28]. Previous studies have primarily compared polyester and PTFE grafts in terms of inflammatory response and arterial stiffness [24,25]. However, data directly linking stent metal type (nitinol vs. stainless steel) to hard outcomes such as mortality remained scarce, and no large-scale or meta-analytic evidence has demonstrated worse prognosis with nitinol-based devices.

To our knowledge, this is the first study to examine mortality in relation to pulsatile hemodynamics together with stent material and stent-graft fabric in AAA patients after EVAR [29]. As previous mentioned, the greater tensile strength of nitinol compared with stainless steel likely explains the greater post-EVAR increase in cf-PWV observed with nitinol stents. By contrast, no material-related differences were seen in cAI or Pb, which reflect peripheral arterial properties, and Pf was comparable, indicating the effect was not due to impaired cardiac function. For stent-graft fabrics, Dacron and PTFE produced similar changes in pulsatile hemodynamic parameters, suggesting that the higher mortality associated with PTFE may not be stiffness-mediated and could involve other mechanisms. Nonetheless, after adjustment for stent type and stent-graft fabric, Δcf-PWV remained the strongest determinant of mortality.

This finding underscores the importance of cf-PWV as a key prognostic marker and highlights the role of arterial stiffness in shaping long-term outcomes after EVAR.

However, in clinical practice, stent framework and graft fabric are integrated. In this study, we did not intend to imply that stent material and graft fabric are independent clinical choices. Rather, we performed material-stratified analyses to explore whether intrinsic material properties may contribute to post-EVAR hemodynamic changes. In this study, Excluder stent-graft (nitinol + PTFE) had a higher 2-year mortality compared with Zenith stent-graft, while Endurant and Zenith had similar mortality rate (Supplementary Fig. 1 and Supplementary Table 2). However, the stent-graft wasn’t an independent predictor after adjustment of confounders in the multivariate Cox regression model (Supplementary Table 3).

### Study limitations

This study has several limitations. First, although the cohort was larger than in previous reports, the sample size may still limit the generalizability of the findings to the broader EVAR population. Second, pulsatile hemodynamics were assessed only at baseline and 1 month, and longer-term measurements could better characterize the trajectory of vascular remodeling and its impact on outcomes. Third, detailed anatomic and clinical determinants of device selection, including iliac morphology, aneurysm angulation, and frailty were not systematically collected for all patients and therefore could not be reliably incorporated into multivariable models. To mitigate confounding, we adjusted for key clinical and aneurysm-related variables that are known to influence both device selection and prognosis, including age, renal function, aneurysm diameter, and baseline cf-PWV. Given the limited number of mortalities, we did not apply propensity score weighting or hierarchical modeling, as these approaches risk model instability and overfitting in this setting. Fourth, the sample size or power calculation was not performed as this study was an observational cohort based on consecutively enrolled patients undergoing EVAR during the study period. The sample size was therefore determined by clinical availability rather than hypothesis-driven enrollment. Nevertheless, the final cohort of 265 patients is larger than most prior studies evaluating pulsatile hemodynamics after EVAR and provided sufficient statistical power to detect clinically meaningful associations between post-procedural changes in cf-PWV and mortality. Further studies with larger, multicenter cohorts, extended follow-up, and more detailed clinical and hemodynamic assessments are warranted to validate these findings and clarify the underlying mechanisms.

## Conclusion

In this study, AAA patients treated with nitinol stents showed a greater increase in cf-PWV, comparing with those receiving stainless-steel stents. Pulsatile hemodynamic changes were broadly similar between Dacron and PTFE stent-grafts; However, the nitinol stents and PTFE stent-grafts was associated with the highest 2-year all-cause mortality, whereas stainless-steel stents with Dacron stent-grafts were associated with more favorable outcomes. After adjusting for confounders, Δcf-PWV remained the strongest independent predictor of 2-year mortality. These findings underscore the prognostic value of post-EVAR arterial stiffening and suggest that device characteristics may influence outcomes indirectly through their effects on vascular hemodynamics. Further prospective studies are required to validate these observations and clarify causal mechanisms.

## Supplementary information

Supplementary Information

## Glossary of abbreviations

AAA: Abdominal aortic aneurysm
ACC/AHA: American College of Cardiology / American Heart Association
cAI: Carotid augmentation index
cf-PWV: Carotid–femoral pulse wave velocity
CI: Confidence interval
CT: Computed Tomography
DBPb: Brachial diastolic blood pressure
eGFR: Estimated glomerular filtration rate
ESC: European Society of Cardiology
EVAR: Endovascular aortic repair
GEE: Generalized estimating equation
HR: Hazard ratio
IRB: Institutional Review Board
IVSd: Interventricular septum thickness in diastole
LAVi: Left atrial volume index
LVEF: Left ventricular ejection fraction
LVIDd: Left ventricular internal diameter at end-diastole
LVIDs: Left ventricular internal diameter at end-systole
LVMi: Left ventricular mass index
MBP: Mean blood pressure
PASP: Pulmonary artery systolic pressure
Pb: Backward pressure wave amplitude
Pf: Forward pressure wave amplitude
PPb: Brachial pulse pressure
PTFE: Polytetrafluoroethylene
LVPWd: Posterior wall (thickness, diastole)
SBPb: Brachial systolic blood pressure
