# Supplementary material for: Impact of stent materials and hemodynamic changes after endovascular aneurysm repair for abdominal aortic aneurysm
Source: Hypertens Res. 2026 Mar 30;49(5):1673–82. doi: 10.1038/s41440-026-02595-8 (PMC13149008; doi:10.1038/s41440-026-02595-8)
Supplement: Supplementary file 1 — Supplementary Information [file 41440_2026_2595_MOESM1_ESM.docx]

**SUPPLEMENTARY MATERIAL**

**Impact of Stent Materials and Hemodynamic Changes After Endovascular Aneurysm Repair for Abdominal Aortic Aneurysm**

Chih-Hsueh Tseng^1,2,3^, Wei-Min Huang^1,2,4^, Hao-Chih Chang^2,5^, Wen-Chung Yu^1,2^, Chun-Che Shih^6^; Chern-En Chiang^2,8,9^, Chen-Huan Chen^2,7,8^, Shih-Hsien Sung^1,2,3,8^

^1^Department of Medicine, Taipei Veterans General Hospital, Taipei, Taiwan ^2^Department of Internal Medicine, National Yang-Ming Chiao Tung University, Taipei, Taiwan

^3^Institute of Emergency and Critical Care Medicine, National Yang-Ming Chiao-Tung University, Taipei, Taiwan ^4^Department of Medicine, Kinmen Hospital, Ministry of Health and Welfare, Jinhu, Taiwan

^5^Department of Medicine, Taipei Veterans General Hospital Taoyuan Branch, Taoyuan, Taiwan ^6^ Institute of Clinical Medicine, School of Medicine, National Yang Ming Chiao Tung University, Taipei, Taiwan, ROC.

^7^Department of Medical Education, Taipei Veterans General Hospital, Taipei, Taiwan ^8^Cardiovascular Research Center, National Yang-Ming Chiao Tung University, Taipei, Taiwan ^9^General Clinical Research Center, Taipei Veterans General Hospital, Taipei, Taiwan

*** Correspondence:**

Shih-Hsien Sung, M.D., Ph.D.

Institute of Emergency and Critical Care Medicine, National Yang-Ming Chiao-Tung University, College of Medicine, Taipei, Taiwan.

No. 155, Sec. 2, Linong Street, Beitou District, Taipei, 112 Taiwan (ROC).

Tel: 886-2- 2821-1699

Fax: 886-2- 2820-2190

E-mail: [mr.sungsh@gmail.com](mailto:mr.sungsh@gmail.com)

| **Supplementary Table 1. Outcomes of 2-year follow-up** | | | | |
| --- | --- | --- | --- | --- |
|  | **All**  **(n=265)** | **Nitinol stent**  **(n=133)** | **Stainless steel stent**  **(n=132)** | **P value^*^** |
| Mortality | 25 (9.4) | 17 (13.5) | 8 (6.0) | 0.042 |
| Cardiovascular death | 11 (4.2) | 7 (5.2) | 4 (3.0) | 0.301^$^ |
| Non-cardiovascular death | 14 (5.2) | 10 (7.5) | 4 (3.0) |  |
| Endoleak | 25 (9.4) | 18 (13.5) | 7 (5.3) | 0.007 |
| Type I | 6 (2.5) | 2 (1.7) | 4 (3.2) |  |
| Type II | 17 (7.0) | 15 (12.8) | 2 (1.6) |  |
| Type III | 1 (0.4) | 0 (0) | 1 (0.8) |  |
| Type IV | 1 (0.4) | 1 (0.9) | 0 (0) |  |
| Reintervention | 9 (3.8) | 3 (2.6) | 6 (4.8) | 0.372 |
| ^*^Chi-square test  ^$^Competing risk analysis of cardiovascular and non-cardiovascular death using the log-rank p | | | | |

| **Supplementary Table 2. Univariate cox proportional analysis of 2-year all-cause mortality** | | | | |
| --- | --- | --- | --- | --- |
|  | **Univariate analysis** | | |  |
| Variable | HR (95% CI) |  | *p* |  |
| Age, 1 SD = 11.5 years | 1.039 (0.996-1.083) |  | 0.077 |  |
| Gender | 1.378 (0.325-5.847) |  | 0.663 |  |
| Diabetes Mellitus | 0.270 (0.037-1.997) |  | 0.200 |  |
| Hypertension | 0.805 (0.302-2.144) |  | 0.664 |  |
| PAD | 0.498 (0.067-3.679) |  | 0.494 |  |
| eGFR, 1 SD = 26.1 ml/min/1.73m | 0.982 (0.967-0.997) |  | 0.021 |  |
| Stent material (Nitinol v.s. stainless steel) | 0.429 (0.185-0.995) |  | 0.049 |  |
| Stent-graft fabric (PTFE v.s. Dacron) | 0.405 (0.185-0.888) |  | 0.024 |  |
| Stent graft (compared to Zenith Flex) |  |  |  |  |
| Excluder | 2.787 (1.155-6.724) |  | 0.023 |  |
| Endurant | 1.519 (0.457-5.046) |  | 0.495 |  |
| Aneurysm diameter, 1 SD = 2.07 cm | 1.134 (1.013-1.270) |  | 0.029 |  |
| Aneurysm length, 1 SD = 2.61 cm | 0.946 (0.674-1.327) |  | 0.747 |  |
| Aneurysm neck length, 1 SD = 17.2 mm | 1.014 (0.996-1.032) |  | 0.121 |  |
| Hostile neck^*^ | 2.162 (0.732-6.931) |  | 0.163 |  |
| **Baseline, pre-EVAR** |  |  |  |  |
| cAI, 1 SD = 17.9% | 1.688 (0.812-3.507) |  | 0.161 |  |
| cf-PWV, 1 SD = 2.9 m/s | 0.483 (0.274-0.851) |  | 0.012 |  |
| Pb, 1 SD = 8.9 mmHg | 1.060 (0.615-1.826) |  | 0.834 |  |
| Pf, 1 SD = 12.3 mmHg | 0.817 (0.491-1.448) |  | 0.489 |  |
| **On treatment, post-EVAR** |  |  |  |  |
| cAI, 1 SD =35.5% | 1.028 (0.472-2.237) |  | 0.945 |  |
| cf-PWV, 1 SD = 3.49 m/s | 2.288 (1.023-5.121) |  | 0.044 |  |
| Pb, 1 SD = 8.1 mmHg | 0.838 (0.394-1.784) |  | 0.647 |  |
| Pf, 1 SD = 13.9 mmHg | 0.490 (0.176-1.364) |  | 0.172 |  |
| **The changes** |  |  |  |  |
| Delta cAI, 1 SD = 22.7% | 1.207 (0.690-2.113) |  | 0.510 |  |
| Delta cf-PWV, 1 SD = 4.0 m/s | 4.358 (1.713-11.087) |  | 0.002 |  |
| Delta Pb, 1 SD = 6.1 mmHg | 0.815 (0.376-1.767) |  | 0.604 |  |
| Delta Pf, 1 SD = 11.9 mmHg | 0.692 (0.304-1.574) |  | 0.380 |  |
| *Hostile neck was defined as aneurysm neck length < 15mm  cAI, carotid augmentation index; cf-PWV, carotid–femoral pulse wave velocity; eGFR, estimates glomerular filtration rate; EVAR, endovascular aneurysm repair; PAD, peripheral artery disease; Pb, backward pressure wave amplitude; Pf, forward pressure wave amplitude; PTFE, polytetrafluoroethylene | | | | |

| **Supplementary Table 3. Cox proportional analysis of 2-year all-cause mortality** | | | | | | | | | | | |
| --- | --- | --- | --- | --- | --- | --- | --- | --- | --- | --- | --- |
|  | **Model 1** | | |  | **Model 2** | | |  | **Model 3** | | |
| Variable | HR (95% CI) |  | *p* |  | HR (95% CI) |  | *p* |  | HR (95% CI) |  | p |
| Stent material (Nitinol v.s. stainless steel) | 0.719 (0.254-2.036) |  | 0.535 |  | 0.535 (0.131-2.188) |  | 0.384 |  | 0.770 (0.253-2.342) |  | 0.646 |
| Stent-graft fabric (PTFE v.s. Dacron) | 0.861 (0.311-2.383) |  | 0.773 |  | 1.275 (0.322-5.052) |  | 0.730 |  | 0.651 (0.218-1.948) |  | 0.443 |
| Stent-graft (compared to Zenith Flex) |  |  |  |  |  |  |  |  |  |  |  |
| Excluder | 2.387 (0.957-5.958) |  | 0.062 |  | 1.027 (0.287-3.677) |  | 0.967 |  | 1.496 (0.456-4.911) |  | 0.507 |
| Endurant | 1.323 (0.393-4.447) |  | 0.651 |  | 1.719 (0.393-7.524) |  | 0.472 |  | 0.914 (0.174-4.813) |  | 0.915 |
| On-treatment cf-PWV, 1 SD = 2.9 m/s | 2.468 (0.989-6.157) |  | 0.053 |  | 3.373 (1.133-10.042) |  | 0.029 |  | 4.888 (0.910-26.249) |  | 0.064 |
| Delta cf-PWV, 1 SD = 4.0 m/s | 5.118 (1.784-14.687) |  | 0.002 |  | 4.011 (1.154-13.950) |  | 0.029 |  | 9.708 (1.002-94.198) |  | 0.049 |
| Model 1: adjusted for age, gender  Model 2 adjusted for age, gender, aneurysm diameter, and baseline cf-PWV  Model 3: adjusted for age, gender, BMI, hypertension, diabetes, hyperlipidemia, stent length, smoking  BMI, body mass index; cf-PWV, carotid–femoral pulse wave velocity | | | | | | | | | | | |

| **Supplementary Table 4. Multivariate analysis of all-cause mortality with all variables** | | | | | | | | | | | |
| --- | --- | --- | --- | --- | --- | --- | --- | --- | --- | --- | --- |
| Variable | **Model 1** | | | | **Model 2** | | |  | **Model 3** | | |
|  | HR (95% CI) |  | *P* |  | HR (95% CI) |  | *p* |  | HR (95% CI) |  | *p* |
| Age, years | 1.131 (0.984-1.299) |  | 0.083 |  | 1.130 (0.979-1.304) |  | 0.094 |  | 0.969 (0.807-1.163) |  | 0.734 |
| Gender | 0.955 (0.126-7.229) |  | 0.964 |  | 0.963 (0.115-8.329) |  | 0.973 |  | 0.956 (0.081-9.446) |  | 0.945 |
| Aneurysm diameter, cm |  |  |  |  | 1.014 (0.956-1.076) |  | 0.638 |  | 1.028 (0.970-1.090) |  | 0.357 |
| Baseline cf-PWV, m/s |  |  |  |  | 0.997 (0.993-1.002) |  | 0.222 |  |  |  |  |
| BMI, kg/m^2^ |  |  |  |  |  |  |  |  | 0.711 (0.456-1.106) |  | 0.130 |
| Hypertension |  |  |  |  |  |  |  |  | 0.245 (0.017-3.444) |  | 0.297 |
| Diabetes mellitus |  |  |  |  |  |  |  |  | 0.944 (0.213-8.533) |  | 0.984 |
| Hyperlipidemia |  |  |  |  |  |  |  |  | 0.854 (0.012-9.413) |  | 0.977 |
| Stent length, cm |  |  |  |  |  |  |  |  | 0.976 (0.936-1.018) |  | 0.263 |
| Smoking |  |  |  |  |  |  |  |  | 2.052 (0.157-26.756) |  | 0.583 |
| Delta cf-PWV, 1 SD = 4.0 m/s | 5.118 (1.784-14.687) |  | 0.002 |  | 4.011 (1.154-13.950) |  | 0.029 |  | 9.708 (1.002-94.198) |  | 0.049 |
| Model 1: adjusted for age, gender  Model 2 adjusted for age, gender, aneurysm diameter, and baseline cf-PWV  Model 3: adjusted for age, gender, BMI, hypertension, diabetes, hyperlipidemia, stent length, smoking  BMI, body mass index; cf-PWV, carotid–femoral pulse wave velocity | | | | | | | | | | | |

**Supplementary Figure 1. Kaplan-Meier analysis of 2-year all-cause mortality in EVAR subjects regarding different stent-grafts**


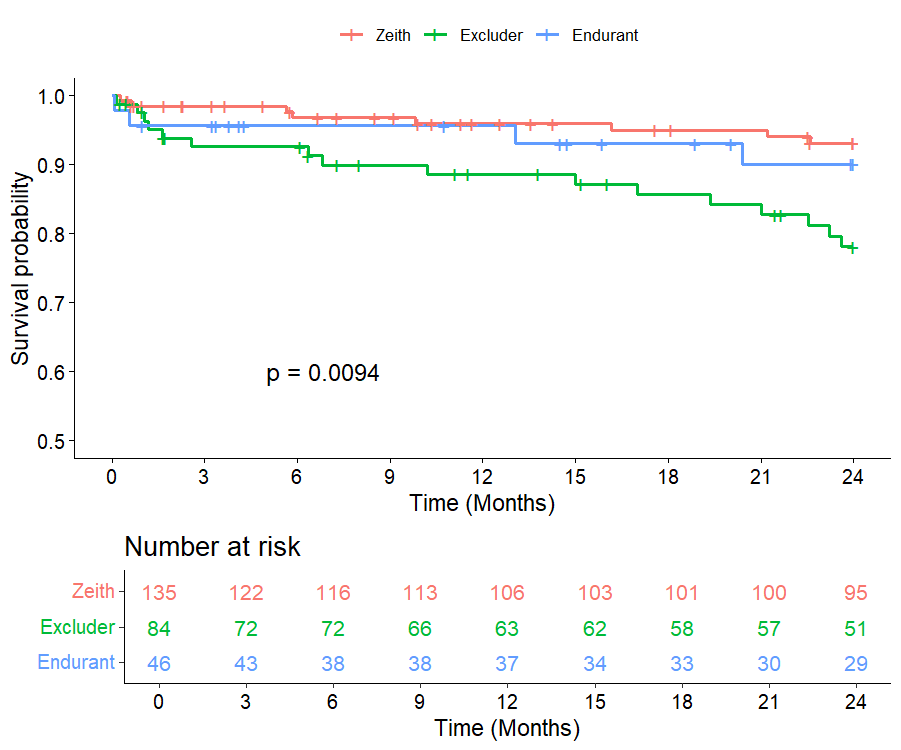


**Supplementary Figure 2. Mediation analysis of delta of cf-PWV and post-EVAR cf-PWV prediction of mortality by different stent materials**


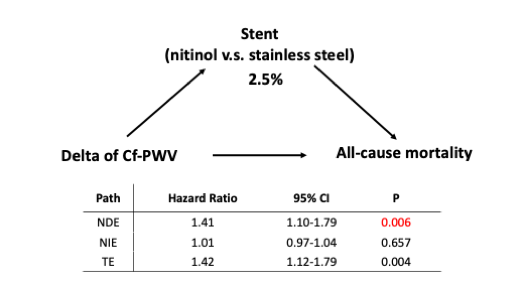

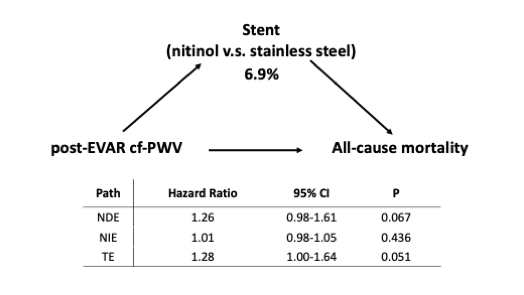


cf-PWV, carotid-femoral pulse wave velocity; CI, confidence interval; NDE, net direct effect; NIE, net indirect effect; TE, total effect
